# Circulating miRNAs as early indicators of diet and physical activity response in women with metastatic breast cancer

**DOI:** 10.2144/fsoa-2020-0208

**Published:** 2021-03-04

**Authors:** Jessica Olson, Patricia Sheean, Lauren Matthews, Christopher R Chitambar, Anjishnu Banerjee, Alexis Visotcky, Marcelo Bonini, Melinda Stolley

**Affiliations:** 1 Medical College of Wisconsin, Milwaukee, WI 53226, USA; 2 Loyola University, Chicago, IL 60660, USA; 3 Northwestern University, Chicago, IL 60208, USA

**Keywords:** biomarkers, breast cancer, lifestyle intervention, metastatic breast cancer, miRNAs, survivorship

## Abstract

Treatments for metastatic breast cancer (MBC) improve survival but often impose prolonged symptom burden. We performed molecular characterization of 84 miRNAs in the circulating serum of women with MBC to explore possible early indicators of intervention response. Expression levels of miR-10a-5p and miR-211-5p were downregulated in nonresponders, but upregulated in responders (miR-10a-5p: 0.40-fold and eightfold; miR 211-5p: 0.47-fold and fourfold). miR-205-5p expression was upregulated in both nonresponders and responders, but to a greater extent in responders (1.8-fold and sixfold). Additionally, levels of miR-10a-5p were negatively correlated with expression levels of IL-6 (r = -0.412). Exploration of these pathways may reveal mechanisms of action in lifestyle interventions aimed at improving quality of life and impacting disease progression for women with MBC.

## Lay abstract

As treatment for women with metastatic breast cancer improves survival rates, interventions are needed that relieve symptom burden. We examined the serum of women with metastatic breast cancer who participated in a lifestyle intervention that improved diet and increased physical activity. Three miRNAs were discovered that may serve as early indicators of the ability of lifestyle interventions to improve quality of life and impact disease progression.

## Tweetable abstract

Three miRNAs may predict how women with metastatic breast cancer respond to lifestyle interventions.

Breast cancer is the second most common cancer in women in the US. Median survival from metastatic breast cancer (MBC), defined as breast cancer found at sites distant from the breast and regional lymph nodes, has increased from 4 to 30% in the last two decades [1–4]. Therapeutic goals of MBC are prolonging life, improving quality of life and symptom palliation [5,6]. Options for treatment depend on the location and morphological characteristics of the metastasis and include radiation, hormone blockade, immunotherapy and chemotherapy [7]. While these treatments typically improve survival, they can result in additional symptoms beyond those imposed by the tumor itself [8].

In diverse populations of early stage breast cancer survivors, there is a positive correlation between healthy lifestyle behaviors, quality of life and breast cancer outcomes [9–12]. To explore this phenomenon, lifestyle interventions targeting diet and physical activity changes are conducted in randomized controlled trial settings. These interventions often use a comprehensive approach that includes dietary, physical activity and behavior modification components [13]. Results of these studies often include improved body composition, enhanced quality of life and improved levels of metabolic and oncogenic risk biomarkers [13–16]. In one such intervention trial, patients with early stage breast cancer who achieved BMI and weight loss goals displayed serum-circulating miRNA expression profiles correlated with suppression of oncogenic cell signaling pathways [17].

miRNAs are small, noncoding RNA molecules that can regulate carcinogenesis and cancer development through their complementary binding to the mRNAs of oncogenes or tumor suppressor genes [18]. miRNAs are well established regulators in the proliferation, migration and self-renewal of cancer cells and stem cells [19]. miRNAs are dysregulated at all stages (I–IV) of breast cancer, and play roles in tumor initiation, progression and treatment response [20–22]. An increasing body of literature is exploring the potential for a panel of miRNAs that are commonly dysregulated in patients with breast cancer to serve as biomarkers for diagnosis and treatment. In 2015, Bertoli *et al.* demonstrated that circulating miRNA profiles have better diagnostic and prognostic performance than individual miRNAs in breast cancer [21]. In 2017, Nassar *et al.* described circulating miRNAs as easily accessible and quantifiable, with strong potential to detect breast cancer at earlier stages and improve patient survival outcomes [20].

Whereas protein biomarkers, such as inflammatory cytokines, appear in serum long after tissue damage has occurred, miRNAs appear in body fluids earlier than conventional biomarkers [23]. Due to their regulatory roles and stable expression in serum, miRNAs are promising targets for detecting cancer, predicting prognosis and targeting treatments [24–26]. The current study examines miRNA expression profiles in a subset of women with clinically stable MBC participating in Every Day Counts, an intervention targeting nutrition and physical activity. We explore the expression patterns of 84 miRNAs on serum inflammatory markers, seeking to identify miRNAs which could aid in discovering markers to differentiate individuals who will or will not respond to a lifestyle intervention. We hypothesize that not only may miRNAs serve as biomarkers to distinguish diagnosis and treatment for breast cancer, but also help guide patients to dietary, physical activity and behaviors that will improve breast cancer outcomes and quality of life.

## Materials & methods

### Parent study description & design

The Every Day Counts pilot study aimed to establish the safety, feasibility and potential efficacy of an intervention promoting the ACS Nutrition and Physical Activity Guidelines in 40 women with clinically stable MBC. Patients were eligible if they were female, over 18 years of age, and had: a confirmed MBC diagnosis, written documentation from their oncologist to participate in the intervention and a life expectancy of over 6 months. In addition, all participants were required to have access to a mobile phone with text messaging, be able to understand and speak English fluently, and be currently identified as being nonadherent to the ACS nutritional or physical activity guidelines for cancer survivors. Participants were randomized to either the intervention or a waitlist-control group. The intervention was conducted over a 12-week period and included weekly lifestyle coaching supporting plant-based eating, 150 min of moderate physical activity and twice weekly strength training. Thirty-five women completed the trial. Body composition, physical functioning, diet, physical activity, physical symptomology, quality of life and serum biomarkers of inflammation were also assessed before and after program participation.

### Blood sample collection & processing

Fasting blood samples were obtained at baseline and postintervention via venous or portacath blood draws. An amount of 60 ml of blood was collected using ethylenediaminetetraacetic acid tubes. Plasma was separated by 10 min of centrifugation at 400×*g* followed by 10 min of centrifugation at 600×g and stored at -80°C.

### Sample selection

Thirty-five participants had evaluable plasma sample at baseline and post-intervention. To demonstrate our ‘proof of concept,’ we compared miRNA profiles of women with reduced inflammatory cytokine levels compared with those whose serum inflammatory markers either remained unchanged or increased following their participation. Given limitations related to the parent trial sample size, we reasoned that looking at opposite ends of the inflammatory profiles would provide insights into potential signature changes. Thus, we categorized this subset of women as ‘responders’ and ‘nonresponders’. Responders included participants who displayed a positive response to the intervention evidenced by decreased levels of IL-6, TNF-α and CRP. Nonresponders were participants demonstrating no change or increases in these biomarkers (Table 1). Change in circulating biomarkers between these groups was statistically significant for TNF-α (p = 0.033) and CRP (p < 0.001), with a strong trend toward a difference in IL-6 (p = 0.056) (Table 2). We then matched responders and nonresponders by age and body mass index at baseline resulting in a sample of 12 (Table 1). Change in circulating biomarkers was statistically significant for TNF-α (p = 0.033) and CRP (p < 0.001), with a strong trend toward a difference in IL-6 (p = 0.056) (Table 2).

**Table 1.** Demographic characteristics of miRNA-evaluated participants.

	Responder (n = 6)	Nonresponder (n = 6)		
Age (years)	55.17 ± 6.82	59.67 ± 10.13		
Race (white/black)	6/0	5/1		
Hispanic (yes/no)	2/4	5/1		
Weight (kg)	81.72 ± 20.81	75.35 ± 13.90		
BMI (kg/m^2^)	27.45 ± 2.45	30.14 ± 3.48		
Time since Stage IV dx (years)	5.3 ± 4.6	3.6 ± 2.5		
*De Novo* MBC (yes/no)	4/2	3/3		
Age at MBC dx (years)	51.50 ± 10.61	57.00 ± 13.23		
ER positive (yes/no)	3/3	5/1		
PR positive (yes/no/missing)	3/3	4/1/1		
HER2 positive (yes/no)	2/4	0/6		
Metastatic sites				
Bone (yes/no)	2/4	3/3		
Liver (yes/no)	1/5	0/6		
Lung (yes/no)	1/5	0/6		
Brain (yes/no)	0/6	0/6		
Other (yes/no)	2/4	2/4		

	Pre-intervention Mean ± SD	Postintervention Mean ± SD	Pre-intervention Mean ± SD	Postintervention Mean ± SD
IL-6 (pg/mL)	5.83 ± 5.66	2.02 ± 1.92	5.07 ± 2.03	4.32 ± 1.82
TNF-α (pg/mL)	6.62 ± 3.47	5.45 ± 3.05	4.76 ± 2.55	4.83 ± 3.13
CRP (mg/L)	6.05 ± 3.88	4.58 ± 3.64	4.04 ± 2.32	4.15 ± 2.91

CRP: C-reactive protein; ER: Estrogen receptor; MBC: Metastatic breast cancer; PR: Progesterone receptor; SD: Standard deviation.

**Table 2.** Change in circulating biomarkers for miRNA-evaluated participants.

Biomarker	Responder (mean ± SD)	Nonresponder (mean ± SD)	p-value
IL-6 (pg/mL)	-4.09 ± 3.85	-0.52 ± 1.23	0.056
TNF-α (pg/mL)	-1.17 ± 0.74	0.21 ± 1.15	0.033^†^
CRP (mg/L)	-1.50 ± 0.99	0.47 ± 0.28	<0.001^‡^

† p < 0.05.

‡ p < 0.01.

CRP: C-reactive protein; MBC: Metastatic breast cancer; SD: Standard deviation.

### RNA isolation & cDNA preparation

Serum RNA was homogenized in QIAzol lysis reagent and the miRNeasy Plasma/Serum Kit (Qiagen, Germany) was used to isolate RNA, according to the manufacturer’s instructions. Briefly, chloroform was added, and samples were centrifuged to separate aqueous and organic phases. The aqueous phase was combined with ethanol to facilitate binding to miRNeasy Mini spin columns. Samples were washed twice in buffer, and RNA was eluted using RNase-free water. Quality of RNA was estimated using 2000/2000c nanodrop spectrophotometry (Themo Scientific, MA, USA). Since there are no endogenously expressed housekeeping genes in circulating serum, *Caenorhabditis elegans* miR-39 mimic was added to each sample before isolation as a spike-in control to normalize and monitor isolation efficiency. The miScript II RT Kit (Qiagen, Germany) reverse transcribed mature miRNA to cDNA in a final reaction volume of 20 μl containing miScript Reverse Transcriptase Mix, 10x miScript Nucleics Mix and 5x miScript HiFlex Buffer. To complete reverse transcription, samples were incubated for 60 min at 37°C followed by 5 min at 95°C.

### Quantitative real-time PCR

Reverse transcription (RT) quality controls were performed to confirm that no genomic DNA was present in the sample and positive PCR controls ensured that reagents were performing correctly. The relative expression levels of 84 miRNAs were analyzed using the Human Serum & Plasma miScript miRNA PCR Arrays (MIHS-106Z; Qiagen, Germany). Each of these 84 miRNAs are abundantly expressed in serum and represent promising candidates for further study. Each of the 84 miRNA assays are lyophilized into single wells, with the remaining wells on the 96-well plate devoted to quality controls. This approach allows for rapid analysis of miRNAs that are differentially expressed between groups, and is both cost effective and less statistically demanding than screening thousands of miRNAs through sequencing [27]. A total of 24 miRNA arrays were run for this experiment: 12 samples from participants categorized as responders and 12 samples from participant categorized as nonresponders (Table 1). One baseline sample and one postintervention sample were compared for each participant. qRT-PCR was conducted using the CFX 96 Real-Time PCR System (Bio-Rad Laboratories, Inc., CA, USA). cDNA was diluted and combined with QuantiTect SYBR Green PCR Master Mix and miScript Universal Primer (Qiagen, Germany), using low-adhesion pipette tips (VWR, PA, USA). Data collected from these experiments defined a threshold cycle number (C_T_) of detection for the miRNAs present in each sample. C_T_ values for all controls were within the manufacturer's recommended range. Raw data was normalized against a global C_T_ mean of expressed miRNAs for each sample. The ΔΔC_T_ method was used to compute miRNAs as a percentage change against baseline expression levels.

### Statistical analysis

Data were expressed as mean ± standard error of the mean unless otherwise stated. For miRNA Arrays, RT-PCR normalization and quality control were performed with the supplied software (https://dataanalysis.qiagen.com/mirna/arrayanalysis.php). Statistical analyses were conducted in SPSS (IBM SPSS Statistics 24, IBM Corp, NY, USA). miRNA expression levels between intervention arms were compared using linear regression analysis. Spearman correlation coefficients were computed to assess the correlation between miRNA expression levels and IL-6, TNF-α and CRP. p-values less than an α level of 0.05 were considered to be statistically significant.

### Pathway analysis

Our research team employed a two-step approach to propose mRNA targets and canonical pathways associated with miRNAs that were differentially expressed between responder and nonresponder samples. Functional characterization of miRNA regulatory roles and controlled pathways were examined using TargetScan. Enriched gene ontology pathways were evaluated with the DIANA-mirPath v.3 online software suite [28]. Ingenuity Pathway Analysis miRNA target filter (Qiagen, Germany) was used to identify mRNA targets that were either experimentally confirmed or predicted with high confidence.

## Results

### Lifestyle interventions show changes in expression levels of miRNAs for women with MBC

**Figure 1.**
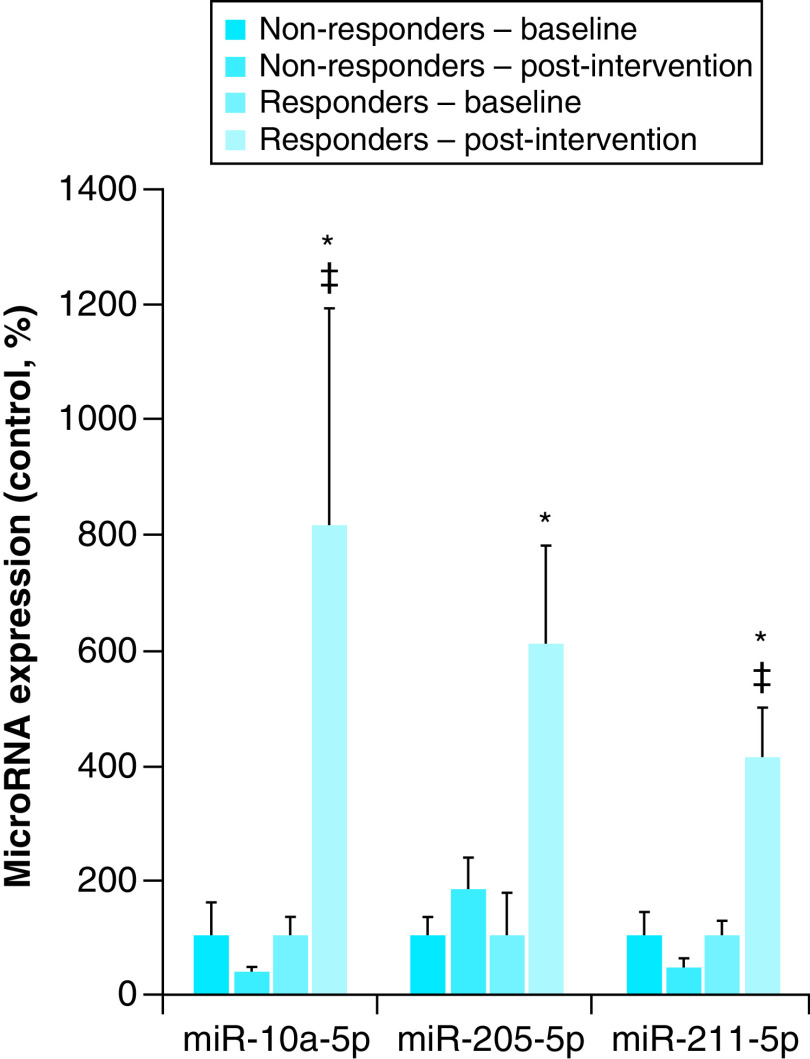
miRNAs are differentially expressed between responder and nonresponder groups following lifestyle intervention participation. Lifestyle intervention participation causes changes in expression levels of miRNAs, as measured by quantitative real-time polymerase chain reaction in participant serum. Nonresponders at baseline (far left, black bars) had significantly lower changes in miRNA expression postintervention (dark gray bar, second from left), than participants categorized as responders (baseline: light gray bar, second from right) and postintervention (white bar, far right). *p-value significantly differentially from baseline measurements. ^‡^p value significantly differentially expressed from postintervention measures in nonresponders. A difference of p < 0.05 was considered statistically significant.

Three miRNAs (miR-10a-5p, miR-205-5p and miR-211-5p) were significantly differentially expressed between responder and nonresponder postintervention groups (p = 0.048, 0.047 and 0.007, respectively). (Figure 1). Eighty-one miRNAs had variable differences in expression within groups, but no significant difference in expression levels between groups (Supplementary Figure 1) [29].

Expression levels of miR-10a-5p were downregulated in nonresponders (0.40-fold ± 0.09), but upregulated in responders (8.14-fold ± 3.74) compared with baseline measurements before participation in the lifestyle intervention (p = 0.048). Expression levels of miR-205-5p were upregulated in nonresponders (1.84-fold ± 0.55) and responders (6.07-fold ± 1.69) but over three-times higher in responders (p = 0.047). miR-211-5p expression levels were downregulated in nonresponders (0.47-fold ± 0.17) but upregulated in responders (4.09-fold ± 0.88) (p = 0.007).

### Expression levels of miR-10a-5p & IL-6 are inversely correlated

**Figure 2.**
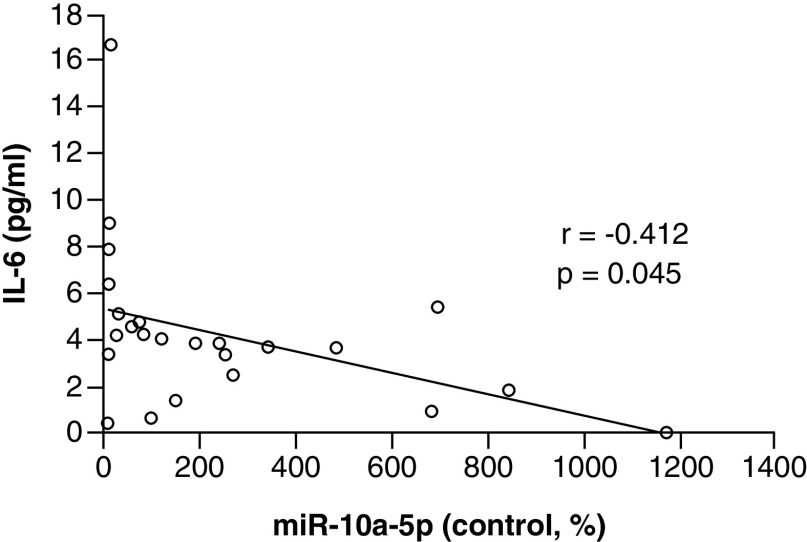
Expression levels of miR-10a-5p are correlated with expression levels of IL-6. Levels of miR-10a-5p and IL-6 were significantly and inversely correlated in samples taken both before and after women with metastatic breast cancer participated in a lifestyle intervention.

Levels of miR-10a-5p were negatively correlated with expression levels of IL-6, suggesting a possible cell signaling pathway mediated by miR-10a-5p. R values were calculated between all three miRNAs and expression levels of IL-6, TNF-α and CRP. All expression levels were showed a trend toward correlation, however, only the relationship between miR-10a-5p and IL-6 were statistically significant (Figure 2).

### Analysis of differentially expressed miRNAs reveal cancer-associated cellular processes & canonical pathways

**Figure 3.**
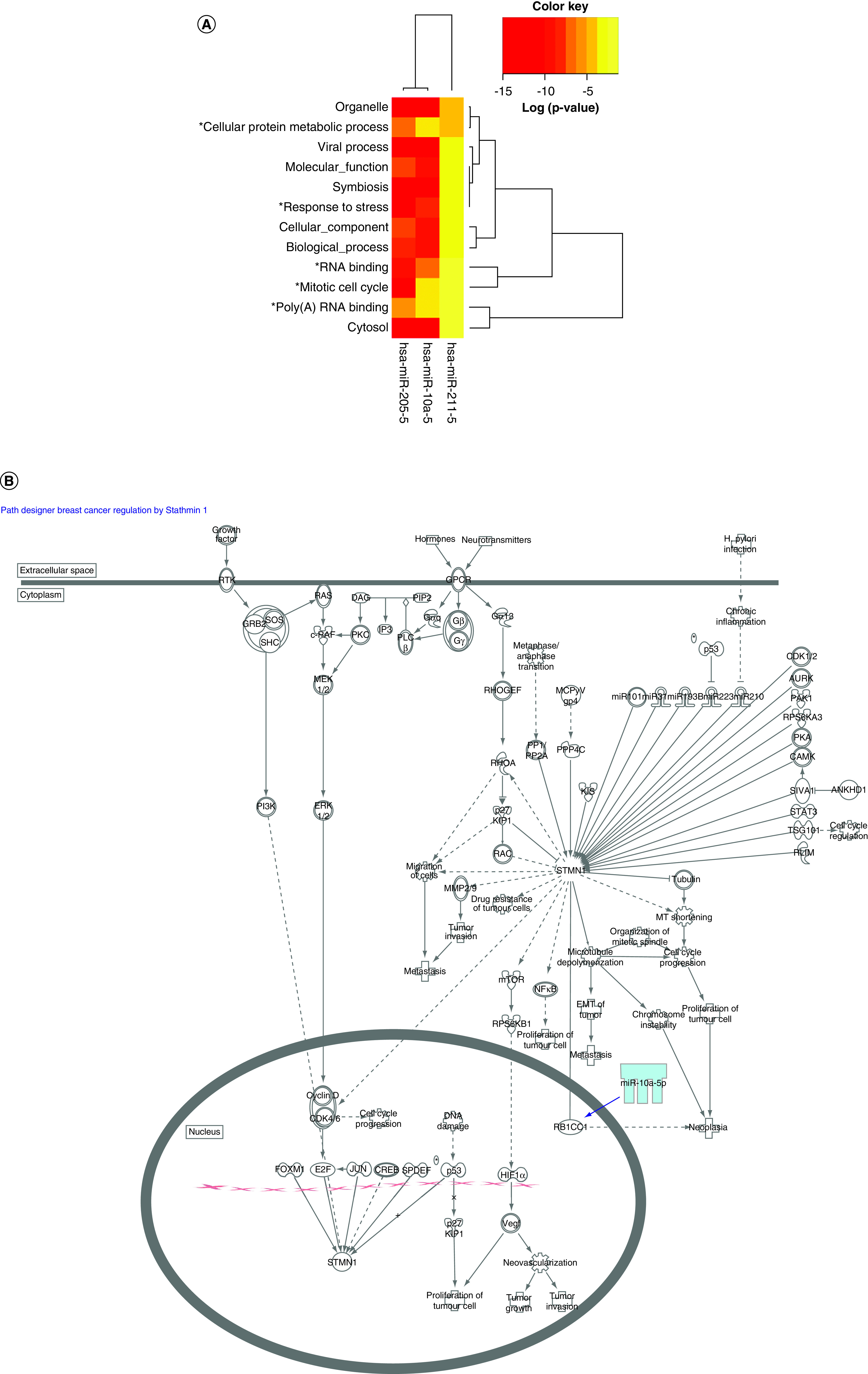
Cellular process and pathway analysis reveals breast cancer associated mechanisms. DIANA TargetScan gene ontology analysis **(A)** and Ingenuity Pathway Analysis **(B)** reveal mechanisms associated with breast cancer metastasis, invasion, motility and regulation. *Processes that are associated with cancer.Figure 3A is adapted from [67] and Figure 3B is adapted from Ingenuity Pathway Analysis Software version 51963813.

Biological pathways regulated by miR-10a-5p, miR-205-5p and miR-211-5p were assessed. DIANA gene ontology analysis revealed mRNA-miRNA interactions that align with basic cellular processes (organelles, viral and biological processes, molecular function, cellular components, cytosol) (Figure 3A). Other mRNA–miRNA interactions align with known tumor associated pathways. For example, oncogenes have known associations to positive regulation of cellular protein metabolic processes, suppression of mitotic cell cycle regulation and can promote cell survival and motility in response to stress [30–32]. RNA binding proteins are associated with drivers of cancer invasion and metastasis and modulate pro-inflammatory cytokine response [33,34]. Ingenuity Pathway Analysis software analysis of canonical pathways for miR-10a-5p revealed only one known pathway: regulation of breast cancer by Stathmin1 (Figure 3B). There were no canonical pathways associated with miR-205-5p or miR-211, breast cancer associated or otherwise.

## Discussion

Understanding the mechanisms of action in lifestyle interventions aimed at improving quality of life and potentially impacting disease progression is critical to improving care of women with MBC. Variable responses to lifestyle interventions appear to depend heavily on genotype, gut microbiota composition and metabolomic expression profiles in healthy, noncancer populations [35–38]. Studies in twins, families and animal models show the interindividual differences in response to interventions that modify diet and exercise behaviors [39]. In women with early stage breast cancer, dysregulation of six miRNAs in cancer-free breast tissue (including miR-205-5p) are linked to clinical outcomes including disease-free interval and overall survival [40]. Hafez *et al.* have shown that eight miRNAs (including miR-10a-5p) correctly identified risk for tumor metastasis in 40 tumor and adjacent non-neoplastic tissue samples from women with early stage breast cancer [41]. In patient serum samples, it is important to note that circulating biomarkers may originate from many cells or tissues in the body, and their presence in serum must be connected to patient outcomes through further experimental studies in models or patient populations. However, previous studies highlight the feasibility of using serum-circulating miRNAs for determining MBC from noninvasive BC, and monitoring disease burden in women with MBC [42–44].

In this study, serum-circulating miR-10a-5p, miR-211-5p and miR-10a-5p were significantly upregulated post lifestyle intervention in responders compared with their baseline and also compared with nonresponders postintervention. These miRNAs are linked to known oncogenic risk pathways and may be potential scalable targets to explain the mechanisms by which lifestyle interventions impact breast cancer progression in women with MBC. For example, serum-circulating CRP, ferritin, B2M, insulin and TNF-α have all been proposed to be potential predictors for progression-free survival and overall survival in women with MBC [45,46].

Upregulation of miR-10a-5p exerts an anti-tumor effect in breast cancer cells by inhibiting the PI3K-AKT pathway and the Stathmin pathway [47]. This miRNA is also negatively correlated with cell proliferation and is decreased in breast cancer tissues and cell lines [47]. In hormone receptor positive breast cancer tissues, downregulation of miR-10a-5p is significantly associated with breast cancer recurrence [48]. Studies involving miR-205-5p show regulation of pathways linked to both negative and positive outcomes for breast cancer patients. Most breast cancer studies conclude that upregulation suppresses tumor growth and metastasis and improves response to chemo- and radiotherapy [49–54].

In a review of the potential for miR-205-5p, Xiao *et al.* note that miR-205-5p expression is lost in both breast tumor tissue and serum, and that the degree of downregulation is an ‘unfavorable clinical prognostic factor’ [49,55]. Within the tumor microenvironment, decreased miR-205-5p expression is linked to stimulation of B cell lymphoma-w-mediated malignant activity and metastatis [50]. In cell lines obtained from a patient with breast cancer at multiple stages of breast cancer progression, miR-205-5p was the only miRNA to exhibit downregulation in correlation with higher histological grades and metastatic potential [51]. A smaller body of evidence suggests that upregulation of miR-205-5p may result in harmful oncogenic effects via increased tumor stage and size, increased metastatic spread and increased incidence of recurrence [56–58]. Yang *et al.* showed that knockdown of miR-205-5p in both breast cancer tissues and cells that resulted in decreased proliferation, migration, invasion and apoptosis in breast cancer cells [56]. In a study by Huo *et al.*, upregulation of miR-205-5p in the serum of women with non-MBC was indicative of tumor recurrence.

To date, only two published studies focus on miR-211-5p and its effects on breast cancer. In a study by Chen *et al.* miR-211-5p was significantly downregulated in both tumor tissue and serum samples from patients with breast cancer and linked to cell proliferation, invasion, migration and metastasis [59]. In an *in vitro* study conducted in breast cancer cell lines, upregulation of miR-211-5p decreased breast cancer cell viability and induced apoptosis [60]. For our study, it is encouraging that both miR-205-5p and miR-211-5p have been investigated in breast cancer tissue, cell lines and serum samples, and are implicated in regulating tumor progression. However, the role of both miRNAs is not established, and our studies may provide novel insight into the potential for miR-205-5p and miR-211-5p to be utilized as biomarkers or potential therapeutic targets.

In 2018, Adams *et al.* investigated changes in serum-circulating miRNA expression profiles associated with obesity and breast cancer recurrence among 100 women with early-stage BC participating in a lifestyle intervention. They discovered several intervention-responsive miRNAs, including miR-10a-5p, potentially contributing to the link between weight gain, body composition measurements (total fat, total mass and total lumbar bone mineral content) and cancer [17]. Together, these studies highlight the importance of identifying serum-circulating miRNAs to serve as biomarkers for lifestyle intervention response. Many mechanistic studies in this field focus on inflammatory cytokines, leptin signaling, insulin resistance and adipokine signaling [61–64]. While these biomarkers provide critical insight into the mechanisms of action by which lifestyle changes impact breast cancer outcomes, upstream indicators such as miRNAs may predict outcomes early on and tailor interventions to maximize participant response. IL-6, for example, is a biomarker indicative of prognosis for relapse-free survival and cancer recurrence [65]. In our study, levels of miR-10a-5p were dramatically increased in responders postintervention and correlated with levels of IL-6. Future studies should consider an examination of miR-10a-5p levels throughout the course of participation in a lifestyle intervention, to determine if an early increase predicts response for participants.

In the setting of MBC, a lifestyle intervention promoting adherence to the ACS Nutrition and Physical Activity Guidelines is a promising method to reduce symptom burden and improve quality of life. However, given the variability in response to diet and exercise in heterogenous populations of MBC survivors, it would be advantageous to identify which women might benefit most. In a 2018 study of advance care planning of women with MBC, patients prioritized ‘managing their physical symptoms, avoiding useless prolongation of dying, having good self-esteem, relieving burdens on family and deepening ties with loved ones’ [66]. Predicting response to lifestyle interventions allows the opportunity to tailor interventions, predict prognosis, maximize intervention response and maintain the priorities of women with MBC.

## Limitations

This investigation provides novel findings with clinical potential, however, inherent limitations deserve mention. First, women in this pilot study were required to be clinically stable; thus, they do not represent the MBC patient population as a whole. Second, the women in the pilot study predominantly self-identified as white, and therefore results cannot be broadly generalized across racial/ethnic groups. Third, the miRNA arrays used in this investigation do not allow for technical triplicates, so variability in samples may be greater than observed upon validation of targets with miRNA assays that allow for multiple samples on a single plate. However, the correlation of our results with known breast cancer-associated pathways lends assurance that our predicted targets are valid candidates for further investigation. Finally, this initial study was conducted in a sample of six responders and six nonresponders. It will be critical for future investigations to both expand the size of the cohort and explore mechanistic links that drive variable response.

## Conclusion

We identified several miRNAs that may serve as a molecular tool of characterization of response to lifestyle interventions for women with MBC. The three miRNAs identified were linked to processes and pathways that regulate oncogenic response and patient outcomes. Future studies will investigate the time course and predictive power of miRNA expression changes in larger cohorts of patients with MBC participating in lifestyle interventions.

## Future perspective

Currently, dietary and physical activity guidelines are a general ‘one-size-fits-all’ recommendation. Our hope is that within 5–10 years, especially for populations like cancer survivors, diagnostic tests will reveal prognostic indicators of response to diet and exercise-based lifestyle modifications and allow for tailored recommendations to maximize response, improve quality of life and relieve symptom burden.

**Summary points**

- The number of women living with metastastic breast cancer has increased as improved treatments increase survivorship, however, this is often accompanied by prolonged symptom burden.
- Lifestyle interventions aim to improve quality of life by improving diet and increasing physical activity.
- In this analysis, circulating miRNA were examined to explore the whether they may serve as early predictors of lifestyle intervention response.
- Three miRNAs, miR-10a-5p, miR-205-5p and miR-211-5p, were significantly differentially expressed between responders and nonresponders.
- In other studies, upregulation of miR-10a-5p exerts an anti-tumor effect in breast cancer cells by inhibiting the PI3K-AKT pathway and the Stathmin pathway.
- In this study, miR-10a-5p was negatively correlated with expression levels of IL-6, suggesting a potential mechanism of action predicting intervention response.
- miR-205-5p and miR-211-5p are also associated with breast cancer progression.
- These miRNAs may serve as a molecular tool for characterization of response to lifestyle interventions for women with metastatic breast cancer.
