# Supplementary figures and images for: Circulating miRNAs as early indicators of diet and physical activity response in women with metastatic breast cancer
Source: Future Sci OA. 2021 Mar 4;7(4):FSO694. doi: 10.2144/fsoa-2020-0208 (PMC8015665; doi:10.2144/fsoa-2020-0208)

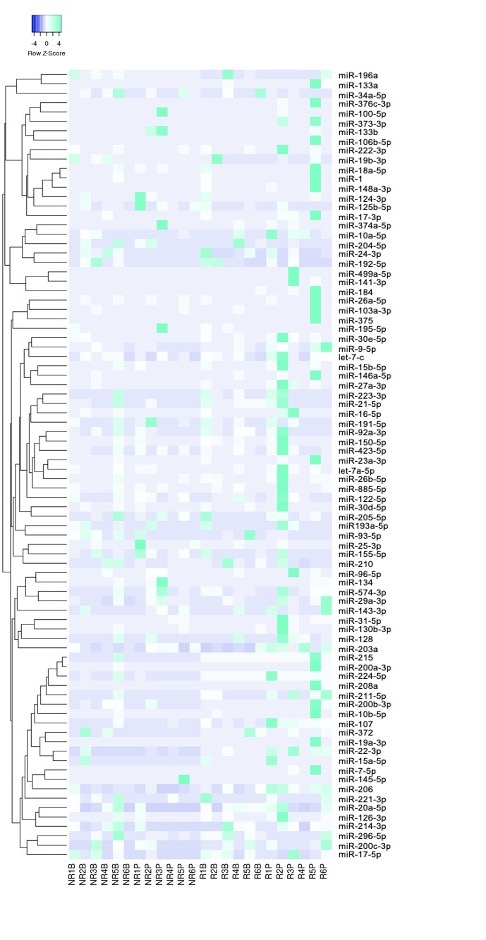

Supplement: Supplementary file 1 [file fsoa-07-694-s1.docx]
